# Biomineralized Composite Liquid Crystal Fiber Scaffold Promotes Bone Regeneration by Enhancement of Osteogenesis and Angiogenesis

**DOI:** 10.3389/fphar.2021.736301

**Published:** 2021-11-08

**Authors:** Yi Zhan, Bing Deng, Huixian Wu, Changpeng Xu, Ruiying Wang, Wenqiang Li, Zhixiong Pan

**Affiliations:** ^1^ Department of Orthopedic Surgery, Affiliated Hospital of Guilin Medical University, Guilin, China; ^2^ Department of Orthopaedics, Guangdong Second Provincial General Hospital, Guangzhou, China; ^3^ Engineering Technology Research Center for Sports Assistive Devices of Guangdong, Guangzhou Sport University, Guangzhou, China; ^4^ Guangxi Health Commission Key Laboratory of Basic Research in Sphingolipid Metabolism Related Diseases, The Affiliated Hospital of Guilin Medical University, Guilin, China

**Keywords:** liquid crystal fiber, biomimetic mineralization, osteogenic differentiation, vascularization, regeneration

## Abstract

Liquid crystals (LCs) are appealing biomaterials for applications in bone regenerative medicine due to their tunable physical properties and anisotropic viscoelastic behavior. This study reports a novel composite poly (L-lactide) (PLLA) scaffold that is manufactured by a simple electrospinning and biomineralization technique that precisely controls the fibrous structure in liquid LC phase. The enriched-LC composites have superior mineralization ability than neat PLLA; furthermore BMSC cells were inoculated onto the HAP-PLLA/LC with hydroxyapatite (HAP) composite scaffold to test the capability for osteogenesis *in vitro*. The results show that the PLLA/LC with HAP produced by mineralization leads to better cell compatibility, which is beneficial to cell proliferation, osteogenic differentiation, and expression of the angiogenic CD31 gene. Moreover, *in vivo* studies showed that the HAP-PLLA/LC scaffold with a bone-like environment significantly accelerates new and mature lamellar bone formation by development of a microenvironment for vascularized bone regeneration. Thus, this bionic composite scaffold in an LC state combining osteogenesis with vascularized activities is a promising biomaterial for bone regeneration in defective areas.

## Introduction

Liquid crystals (LCs) are ubiquitous in our daily lives (Palffy-Muhoray, 2007) and are intrinsically linked to many biological processes (Rey, 2010). Simultaneous cholesteric phase LC is the most common LC tissue in living organisms (Mitov, 2017). Biological research has indicated that the physical requirements of cell membranes are very similar to those of the LC state (Jewell, 2011). In particular, the surface of the cell membrane, which often is in contact with blood, is in a state of a flowing lipid LC because this type of anisotropic viscoelastic material may be a soft elastic solid, which makes the LC potentially useful for engineering the interfaces of living cells (Lockwood et al., 2006). Therefore, various ordered structures in living tissue are analogs of those in the LC phase, involving a variety of biological functions (Satiat-Jeunemaitre, 1992; Charvolin and Sadoc, 2012). In fact, LC materials have potential applications in the biological field because they can form self-assembled structures through specific interactions of noncovalent bonds, making them compatible with materials in living systems (Hamley, 2010). However, polymer LCs are difficult to mold and cannot be used directly as scaffold materials, so compositing polymer substrate and LC is the best way to solve this problem.

Since the extracellular matrix is mainly a fibrous network structure, its structural protein fiber diameter is 50–500 nm (Barnes et al., 2007; Huang et al., 2019a; Hu et al., 2020). Therefore, the fiber network structure of such extracellular matrix can be prepared relatively easily by electrospinning technology, and the bone tissue-engineering scaffold can be further constructed. Simultaneously, hydroxyapatite (HAP) minerals have good biocompatibility, high osteoconductivity, and osteoinductivity that promote osteoblast differentiation and maturation, and are used for repair after bone tissue injury. To prepare a bone repair material with a structure and composition similar to that of human bone, the bone repair material is obtained by inducing mineralization on a biomimetic LC polymer material to form an apatite mineral through a biomineralized method. Ha et al. (2018) modified the orientation mineralization of HAP crystals by soaking them in simulated body fluids (SBFs) *in vitro* to obtain HAP@PTL materials. *In vitro* cell experiments and animal experiments show that HAP@PTL materials have excellent cell compatibility and bone conduction properties.

**SCHEME 1:**
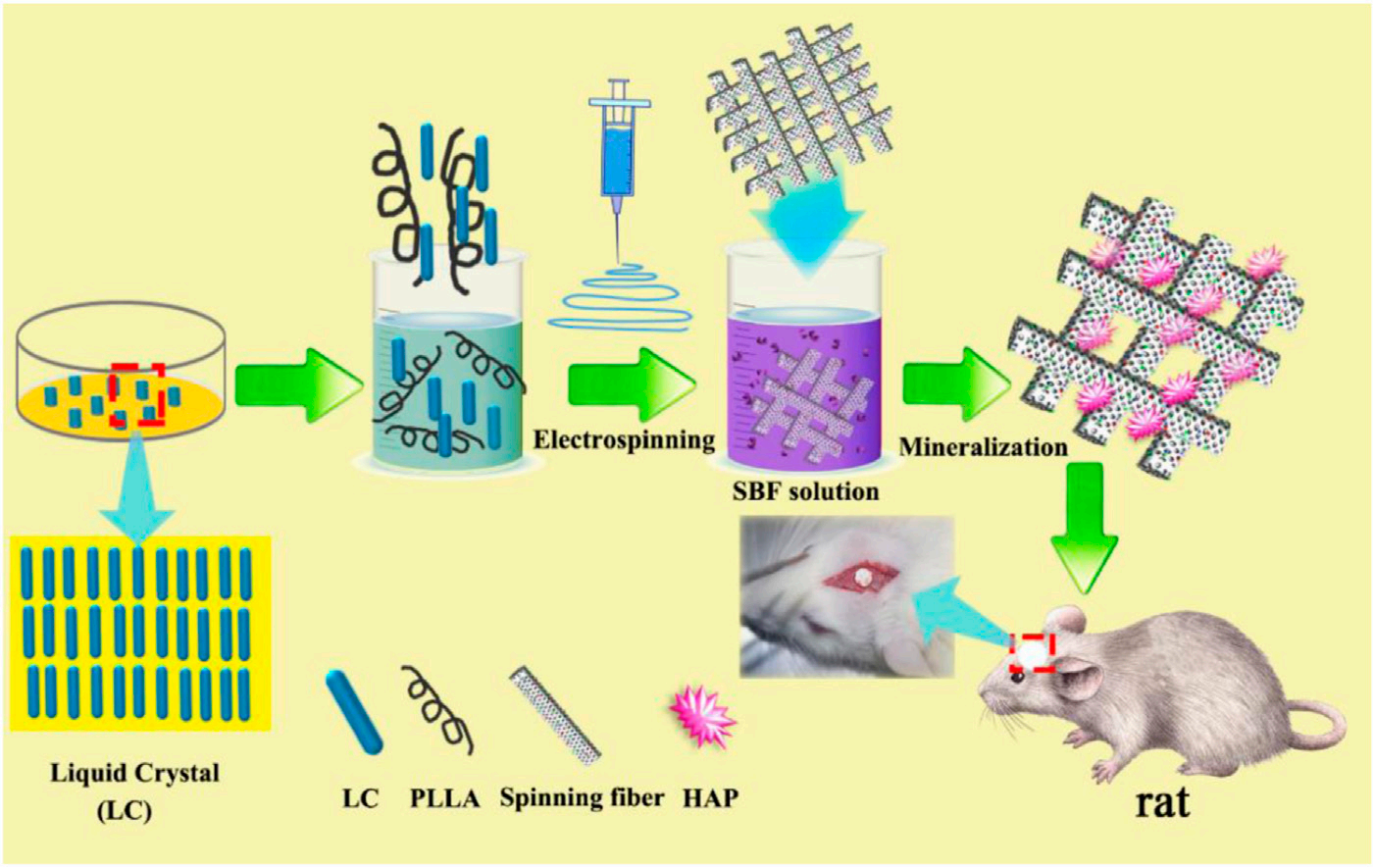
Schematic illustration of the experimental approach of using biomineralized hydroxyapatite (HAP)-poly (L-lactide) (PLLA)/liquid crystal (LC) nanofiber scaffold for promoting calvarial defect.

All tissues and organs in the human body are in the LC state. Herein, the biomaterials with LC state are applied to serve as a template material initially to explore the impact on their mineralization process and biological properties. The strategy combining osteogenic and vascularizatic activities for enhanced bone regeneration *via* a bionic composite fiber scaffold are investigated (Scheme 1). First, the LC polymer was synthesized, and PLLA/LC fiber composite membrane was prepared by electrospinning; then, the mineralization ability of the PLLA and PLLA/LC fiber membranes was studied by an *in vitro* simulated mineralization method. Finally, the physical and chemical properties of the prepared PLLA, PLLA/LC, and HAP-PLLA/LC fiber materials were analyzed and characterized. Furthermore, the cytocompatibility and osteogenic activity of the resulting composite scaffolds were analyzed by the culture of BMSCs cells *in vitro* and animal experiments. This study developed HAP-PLLA/LC fiber materials with excellent angiogenic properties and osteogenic activity. Based on such studies, the synergistic effects of the LC and hierarchical HAP on the osteogenesis and angiogenesis functions of the PLLA-based fibrous membrane can be expected to be a versatile bone tissue-engineering scaffold.

## Materials and Methods

### Materials

Medical-grade PLLA (*M*η = 1 × 10^5^ g/mol) was supplied by Daigang Biomaterial Co. (Jinan, China). Cholesterol (purity ≥ 99.0%) was purchased from Aiwang Chemical Technology Co. (Shanghai, China). Undecylenic acid, chloroplatinic acid (H_2_PtCl_6_▪6H_2_O), and polymethylhydrogensiloxane (PMHS, *M*n = 697) were purchased from Shanghai Macklin Biochemical Co. (Shanghai, China). Dicyclohexylcarbodiimide (DCC), 4-dimethylaminopyridine (DMAP), N,N-dimethylformamide, toluene, dichloromethane (DCM), hydrochloric acid (HCl), sodium hydroxide (NaOH), sodium bicarbonate (NaHCO_3_), anhydrous magnesium sulfate (MgSO_4_), methanol, and ethanol were supplied by Sinopharm Chemical Reagent Co. (Shenyang, China). SBF (1.5 × SBF) was obtained from Xinhua Luyuan Technology Co., Ltd. (Beijing, China).

### Preparation of the Liquid Crystal

**FIGURE 1:**
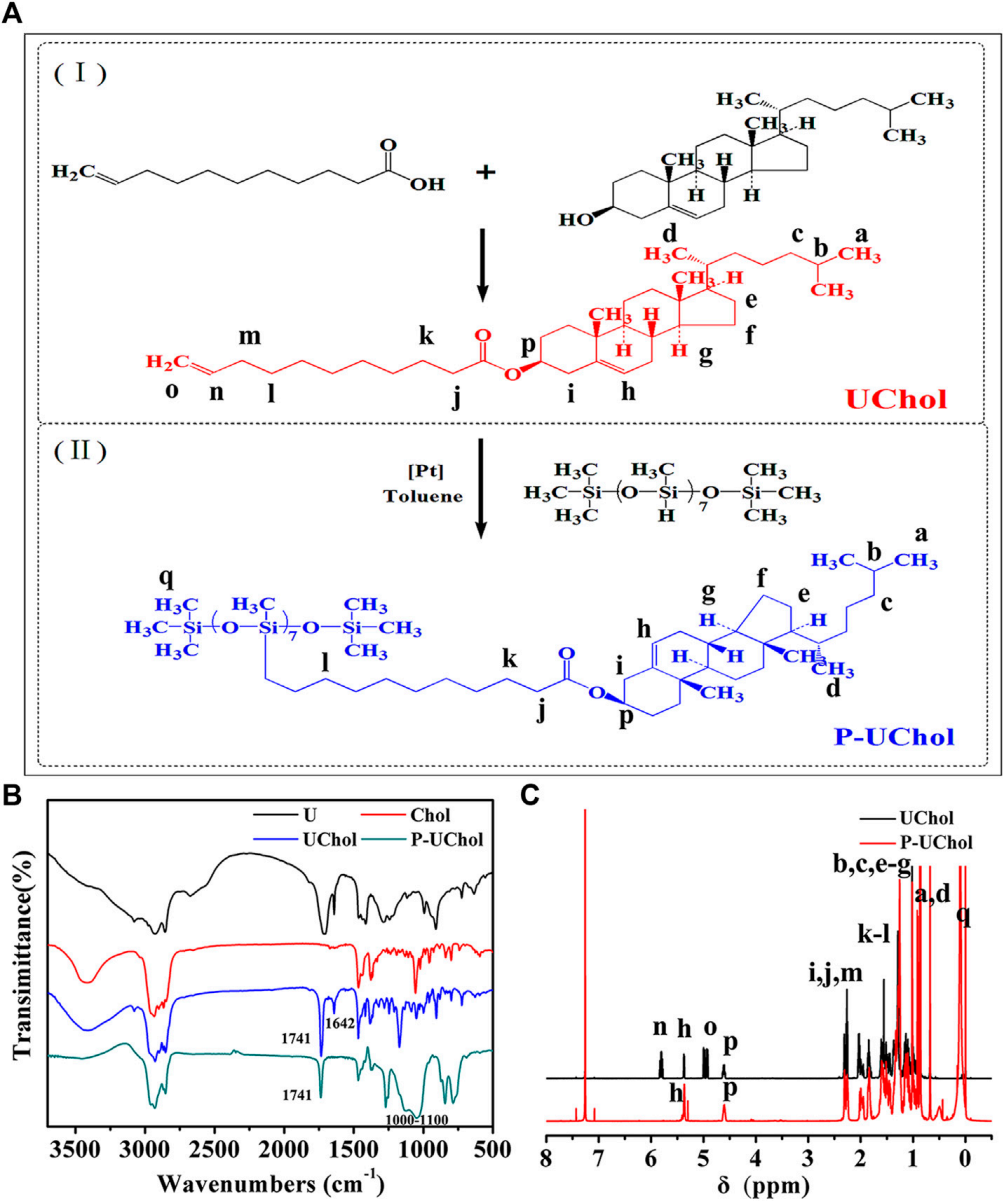
**(A)** Synthetic route of the liquid crystal (LC); **(B)** Fourier transformation infrared (FT-IR), and **(C)** ^1^H-magnetic resonance (NMR) spectra of LC compounds.

The synthetic route used to obtain the LC compounds is shown in Figure 1A. First, the undecylcholesteryl ester monomer LC is prepared by a simple esterification reaction, followed by hydrosilylation to obtain the side chain polymer LC. The specific experimental method is as follows:

Cholesterol (0.02 mol) was dissolved in 100 ml of anhydrous DCM, and DMAP (0.0012 mol) and undecylenic acid (0.03 mol) were added to the above solution while stirring; finally, the dehydrating agent DCC (0.03 mol) was added. The reaction was stirred at 30°C for 20 h in the dark, and the urea formed by the absorption of DCC was removed by filtration. The filtrate was washed successively with distilled water, NaOH solution, NaHCO_3_ solution, and distilled water and then dried over anhydrous magnesium sulfate overnight. After filtration, the DCM solvent was evaporated under normal pressure to obtain a pale yellow material. Pure cholestearyl undecanoate LC (UChol) was obtained by recrystallization from ethanol.

The above monomer and PMHS were dissolved in toluene, and a small amount of H_2_PtCl_6_⋅6H_2_O was added under reflux for 24 h at 65–70°C. At the end of the reaction, the reaction solution was poured into methanol to induce precipitation, and the precipitate was filtered and washed with hot methanol under suction several times to obtain the side chain polymer LC (P-UChol).

### Preparation of the poly(L-Lactide)/Liquid Crystal Fiber Composites and Mineralization

The preparation of the PLLA/LC fiber composites was performed according to the electrospinning method reported in previous studies (Chen et al., 2019a). First, PLLA (0.7 g) and a specific amount of LC (including 0.07, 0.14 and 0.21 g) were, respectively, dispersed in a 50 ml Erlenmeyer flask with 7 ml of DCM to dissolve the PLLA under magnetic stirring until the solution became clear and transparent. Then, 3 ml of N,N-dimethylformamide solvent was added, and stirring was continued for 12 h. Finally, a PLLA/LC electrospinning mixed solution with different LC contents was prepared.

The electrospun fiber materials were prepared by the following method: The above-described PLLA/LC mixed solution with a concentration of 7% was prepared. In composite fibers, the mass ratios of LC are 10, 20, and 30%, respectively. The spinning equipment was continuously spun at a rate of 1 ml/h at a voltage of −2 to 9 kV for 6 h, and the roller speed was 600–1,000 r/min.

The prepared spun fiber material was placed in a 500-ml plastic beaker, and then 500 ml of 1.5 × SBF solution was poured into the beaker. The solution was placed in a 37°C constant temperature water bath for mineralization and covered with a plastic wrap. The 1.5 × SBF solution was prewarmed to 37°C and changed every other day during mineralization. After 7 days, the spun fiber materials were removed, washed with deionized water, and dried in a vacuum oven for 24 h to obtain the mineralized films, including HAP-PLLA, HAP-10% PLLA/LC, HAP-20% PLLA/LC, and HAP-30% PLLA/LC.

### Characterization of the Liquid Crystals and poly(L-Lactide)/Liquid Crystal Composites

The attenuated total reflectance-Fourier transformation infrared (ATRFTIR, Perkin Elmer, Germany) was performed to the chemical structure of composites.

^1^H magnetic resonance (^1^H-NMR) spectra were recorded with a Bruker 500 MHz Ascend high-resolution NMR spectrometer (Bruker, Karlsruhe, Germany), and the chemical shifts were measured by using tetramethylsilane (TMS) as an internal standard.

Differential scanning calorimetry (DSC) analysis was performed with a Discovery 25 DSC calorimeter (TA, Massachusetts, United States) with a heating and cooling rate of 10°C/min under a nitrogen atmosphere.

The LC texture of the samples was observed and recorded with polarizing optical microscopy (POM) (Leica DMRX, Wetzlar, Germany) with a Linkam THM5600 hotstage.

The surface topography of the PLLA fibers and the mineral was investigated by a field emission scanning electron microscope equipped with an energy dispersive spectrometer (FE-SEM, EDS, ULTRA 55, Carl Zeiss, Germany). The fiber diameter and pore size was calculated by ImageJ software.

The crystallization behavior of deposited minerals was measured by x-ray diffractometer (XRD, Dmax-1200, Japan Rigaku) with Ni-filtered Cu-Ka ray line.

Tensile performance was measured on a universal mechanical testing machine (AG-1, SHIMADZU, Japan) with a crosshead speed of 5 mm/min. Static contact angle measurements were carried out *via* a DSA100 (KRÜSS, Germany) Drop Shape Analysis System goniometer to evaluate the hydrophilicity of the fiber scaffolds. The contact angle (CA) values were obtained by averaging the values of three parallel experiments for each sample at room temperature.

The *in vitro* degradation performance of resulting samples was measured by weighing approach. Briefly, 0.1 g of the fiber membranes were immersed in a container filled with 10 ml of simulated body fluid (SBF), sealed, and left to degrade in an incubator at 37°C. Samples were taken out every 3 days, washed gently with distilled water, dried, and weighed to calculate the mass loss.

### Characterization of Cellular Behaviors on the Surface of the Composite Scaffolds

PLLA, PLLA composite fiber with an LC content of 30% (denoted PLLA/LC), and PLLA composite fiber with an LC content of 30% after mineralization (denoted HAP-PLLA/LC), with the highest mineralization capacity, were used for the cell experiments. BMSC cells were incubated with basal high-glucose DMEM supplemented with 10% fetal bovine serum (FBS) and 1% penicillin−streptomycin solution in 37°C humidified condition with 5% CO_2_ atmosphere. The culture medium was changed every 2 days. Cytotoxicity and proliferation were measured by an acridine orange/ethidium bromide (AO/EB) staining assay (viability/cytotoxicity) after 1, 3, and 5 days of cell culture. The cytoskeleton of BMSC cells grown on the three nanofiber types (PLLA, PLLA/LC, and HAP-PLLA/LC) was examined using a laser scanning confocal microscope (LSCM). Each cell containing material was gently washed twice with PBS solution and then fixed in a 4% paraformaldehyde solution for 15 min. Subsequently, the cells were washed with 0.1% Triton X-100 for 10 min and blocked with 3% bovine serum albumin (BSA) for 30 min, and then immune stained with rhodamine-conjugated phalloidin (Life Technologies) for F-actin filament and 4′, 6-diamidino-2-phenylindole (DAPI, Life) for nucleus under dark condition. Finally, the sample was mounted on a microscope slide and observed by LSCM. Moreover, the cellular proliferation was further investigated by real-time cell analysis (RTCA) systems (iCELLigence, ACEA Biosciences Inc.). The cell proliferation behavior on the nanofiber scaffolds and the expression of Ki67 proteins were further studied by Western blotting analysis. Blots were performed in triplicate, and band density was quantified by Quantity One software (Bio-Rad).

For osteogenesis study *in vitro*, the alkaline phosphatase (ALP) activity and mineralization of cells cultured for 14 days were detected by the ALP kit and alizarin red staining (ARS) kit (Cyagen, Guangzhou, China) according to the standard instructions. Every test was repeated five times for each specimen. In addition, after 7 and 14 days of culturing, the expression of the osteogenic gene runt-connected transcription factor-2 (Runx-2), alkaline phosphate (ALP), and osteocalcin (OCN) in BMSC cells cultured on the different materials and the expression of the angiogenic gene platelet-endothelial cellular adhesion molecule-1 (PECAM-1/CD31) were analyzed by real-time quantitative PCR (RT-PCR). For the RT-PCR experiment, the isolation of total RNA was performed using TRIzol reagent (Invitrogen), and reverse transcription of the complementary DNA (cDNA) was performed with the PrimeScript RT reagent kit (Takara Bio Inc., Shiga, Japan). RT-PCR was performed using a SYBR Green qPCR kit (Invitrogen) on a 7,500 real-time PCR system. The amplification of the cDNA sample was carried out on a PCR instrument according to a previously described procedure (Chen et al., 2019b; Zhang et al., 2020). The relative quantification of the normalized β-actin and GAPDH antibody was used as an internal reference, and the expression of the genes was determined using the 2^−ΔΔCT^ method. The PCR primer sequences are shown in Table 1.

**TABLE 1:** Primer sequences used for real-time-quantitative PCR (RT-qPCR).

Primer	Forward primer (5′–3′)	Reverse primer (5′–3′)
Runx-2	TGC​CCA​GTG​AGT​AAC​AGA​AAG​AC	CTC​CTC​CCT​TCT​CAA​CCT​CTA​A
ALP	CGC​TAT​CTG​CCT​TGC​CTG​TA	GGT​TGC​AGG​GTC​TGG​AGA​AT
OCN	AGG​GCA​ATA​AGG​TAG​TGA​A	CGT​AGA​TGC​GTC​TGT​AGG​C
CD31	GCT​GTC​ACT​GTC​CCC​TAA​GA	GTT​AGG​CAA​AGG​CTG​AAG​CT
β-Actin	GCT​TCT​AGG​CGG​ACT​GTT​AC	CCA​TGC​CAA​TGT​TGT​CTC​TT
GAPDH	GGGAAACTGTGGCGTGAT	GAG​TGG​GTG​TCG​CTG​TTG​A

Note. Runx-2, runt-connected transcription factor-2; ALP, alkaline phosphate; OCN, osteocalcin.

### Animal Experiments

This study used 60 healthy Sprague–Dawley rats (7–8 weeks). The average weight of the rats was 200–220 g. All animal experiments were approved by the Ethics Committee of Guilin Medical University (Approval No. 2020-0002). General anesthesia was performed by intramuscular injection of 40 mg/kg of pentobarbital sodium. Local anesthesia was performed in the skull area with 2% lidocaine and adrenaline. A longitudinal incision was made in the sagittal midplane, and a subperiosteal craniotomy was performed to examine the surroundings of the skull and expose the skull. Bone defects were produced by saline and saline flushing. A circular bone hole with a 5-mm diameter was constructed. Then the skull defects were transplanted with the four components (control group, PLLA, PLLA/LC, and HAP-PLLA/LC composite scaffold); the control group was not implanted with any material. After the transplant, the muscles and skin were sutured with sutures. Gentamicin (1 mg/kg) and pyridine (0.5 ml/kg) were injected intramuscularly three times a day. Five rats in each group were sacrificed at 4, 8, and 12 weeks after surgery. Specimens were fixed in 10% formalin. Histological analysis was performed to assess new bone formation.

### Micro-CT and Organizational Morphology Analysis

The regenerated bone tissue in the skull defect area was scanned by micro-CT (Skyscan 1176, Kontich, Belgium) after 12 weeks. Scanned images were reconstructed in 3D with mimics 18.0 software and parameters. Bone mineral density (BMD), bone volume and total volume (BV/TV) in the defect were measured by CTAn program (Skyscan Company).

Specimens were decalcified and embedded in paraffin. The central portion of the implant and defect was cut into 5-μm-thick sections and stained with hematoxylin and eosin (H&E) and Masson’s trichrome stain (MT).

### Identification of Regenerated New Bone by Immunohistochemistry

The human origin of the engineered bone construct after implantation *in vivo* was detected at 4, 8, and 12 weeks using mouse monoclonal antibodies against BMP-2, OCN, and CD31 (Abeam, Cambridge, MA, United States). Tissue sections were deparaffinized with xylene and rehydrated with a series of ethanol washes. The epitope was retrieved by incubation in citrate buffer at 70°C for 40 min, and endogenous peroxidase activity was blocked with 3% H_2_O_2_. The slides were then blocked with 1% BSA for 30 min to reduce nonspecific staining and stained with primary antibody (1:50) overnight in a humid environment. The specimen was then incubated with an anti-mouse IgG secondary antibody (1:500) for 30 min at 37°C. After incubation, streptavidin-HRP and diaminobenzidine (DAB) substrates were added, and the specimens were counterstained with hematoxylin solution.

### Statistical Analysis

Statistical significance was determined by applying Student’s t-test or by a one-way ANOVA followed by Student–Newman–Keuls test using Sigma Stat version 3.5. In this study, *p*-values lower than 0.05, 0.01, or 0.001 were considered statistically significant.

## Results and Discussion

### Synthesis and Structural Characterization

The FT-IR spectra of the monomer UChol and the polymer P-UChol are shown in Figure 1B. The monomer UChol and polymer P-UChol can be obtained by simple esterification and hydrosilylation reactions, respectively. The peak of C = C appears at 1,642 cm^−1^, and the characteristic absorption peak of the carbonyl group appears at 1,741 cm^−1^ in the blue curve, indicating that the hydrogen in Chol and undecylenic acid underwent an esterification reaction, which implied that the UChol monomers had been generated. The C=C peak of the undecylenic acid chain terminal group at 1,642 cm^−1^ disappeared, and the broad Si–O–Si multiple absorption peak of the polymers at 1,000–1,100 cm^−1^ can be observed in the green curve, which confirmed that the Si–H group of the polymer and the C=C of the monomer UChol had completely reacted. Simultaneously, according to the ^1^H-NMR spectrum in Figure 1C, the new signals appearing at 4.98 and 5.80 ppm were attributed to the proton peaks (o and n) caused by the C=C of the end group of undecylenic acid. The characteristic peak of methyl hydrogen on the terminal carbon appears at about 0.91 ppm, the characteristic peak of methylene on the main chain appears at about 1.25 ppm, and the characteristic hydrogen on the ring carbon connected to the newly formed ester group appears at about 4.60 ppm. The above resonances at 4.98 and 5.80 ppm disappeared, and a new peak in siloxane backbone at about 0.21 ppm appeared in the curve for P-UChol, and the characteristic peak of monomer also appears in polymer at the same time, which indicates the formation of the polymer LC. Simultaneously, the analysis results of monomer elements (Elem. Anal.: C: 51.66, H: 11.54, O: 5.83) and polymer elements (Elem. Anal.: C: 66.68, H: 11.54, O: 7.83, Si: 10.54) were provided, which further confirmed the purity of synthetic material. Thus, the above FT-IR and ^1^H-NMR results are consistent with the molecular design.

### Characterization of the Liquid Crystal Properties

The LC properties of the monomer and polymer were characterized by POM and DSC. The physical properties of the thermotropic LC were observed by POM, such as the optical textures and orientation defects. DSC was used to observe the phase transition temperatures, including the glass transition temperature (T_g_), melting temperature (T_m_), and isotropic temperature (T_i_).

**FIGURE 2:**
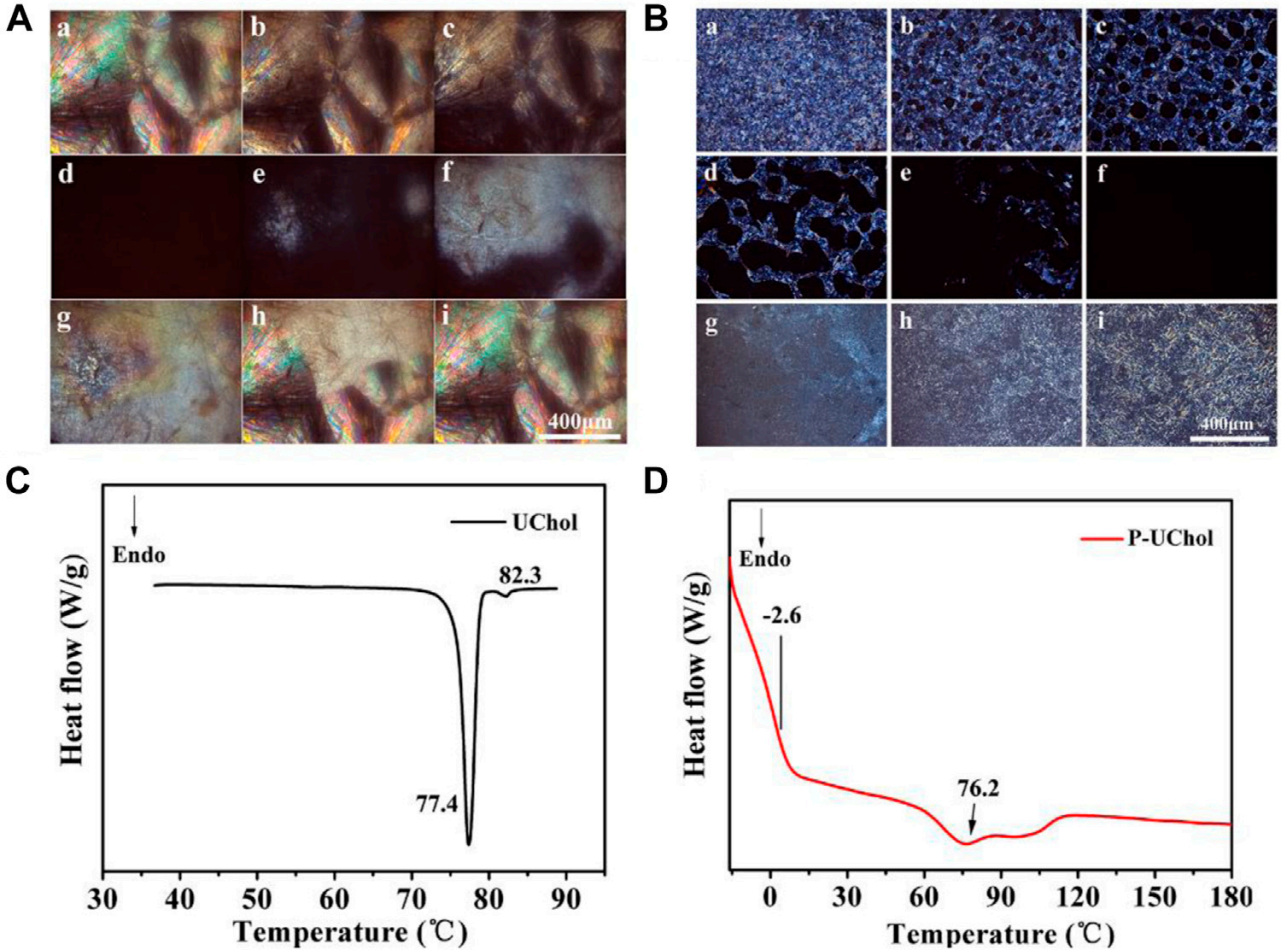
Optical texture of **(A)** monomer cholestearyl undecanoate LC (UChol) and **(B)** polymer side chain polymer LC (P-UChol) changing at different temperatures, a: 50.0°C, b: 76.8°C, c: 77.4°C, d: 81.5°C, e: 72.2°C, f: 70.4°C, g: 64.3°C, h: 50.1°C, i: room temperature. **(C,D)** Differential scanning calorimetry (DSC) thermal curves of the UChol monomer and polymer LC.

According to the POM observation results, the monomer and polymer LC showed the typical color and texture at room temperature. Monomers exhibited crystallization phenomena, but polymer LCs presented the focal conic textures typical of cholesteric LCs. At the same time, the optical texture of LC exhibited different textures at different temperatures, and the texture change in LC during the heating and cooling cycles is shown in Figures 2A,B.

The phase transition temperatures of the LC from DSC curves are summarized in Table 2. The monomer exhibited a sharp melting peak at 77.4°C and a clearing point temperature of 82.3°C. The temperatures described above are consistent with the temperatures exhibited during the change in the LC texture seen by POM described in the supporting material. The polymer LC did not have a melting point but showed a glass transition at −2.6°C, so it was an amorphous compound and exhibited an LC state at room temperature and at the temperature of the normal human body. Two overlapping peaks appeared on the heating curve, while no change in the phase texture appeared in the process of POM observation; therefore, it was considered as only one peak, representing the transition of the cholesteric phase to the isotropic phase at approximately 76.2°C. When the polymethylhydrogensiloxane-grafted monomer was obtained, polymers with different molecular weights were obtained due to the different grafting rates, resulting in a slight deviation in the clearing point and the formation of overlapping peaks. Similar to the results shown in Figures 2B–F, the polymer LC shows a completely dark field of view when heated to 76.8°C, and the optical texture of the LC reappears again after cooling.

**TABLE 2:** Differential scanning calorimetry **(**DSC) and polarizing optical microscopy (POM) results for the monomer and polymer liquid crystal (LC).

Monomers/polymers	DSC				POM	
	T_g_ (°C)	T_m_ (°C)	T_i_ (°C)	△T (°C)	T_cl_ (°C)	T_lc_ (°C)
UChol	—	77.4	82.3	4.9	81.5	72.2
P- UChol	−2.6	—	76.2	78.8	76.8	68.2

Note. △T, the mesogenic phase temperature ranges (i.e., T_i_–T_m_; T_i_–T_g_); T_cl_, the temperature at which the texture completely disappears when heated under POM; T_lc_, the temperature at which the texture reappears when cooled under POM; UChol, cholestearyl andecanoate LC; P-UChol, chain polymer LC.

### Characterization of the poly(L-Lactide)/Liquid Crystal Composites and its Minerals

**FIGURE 3:**
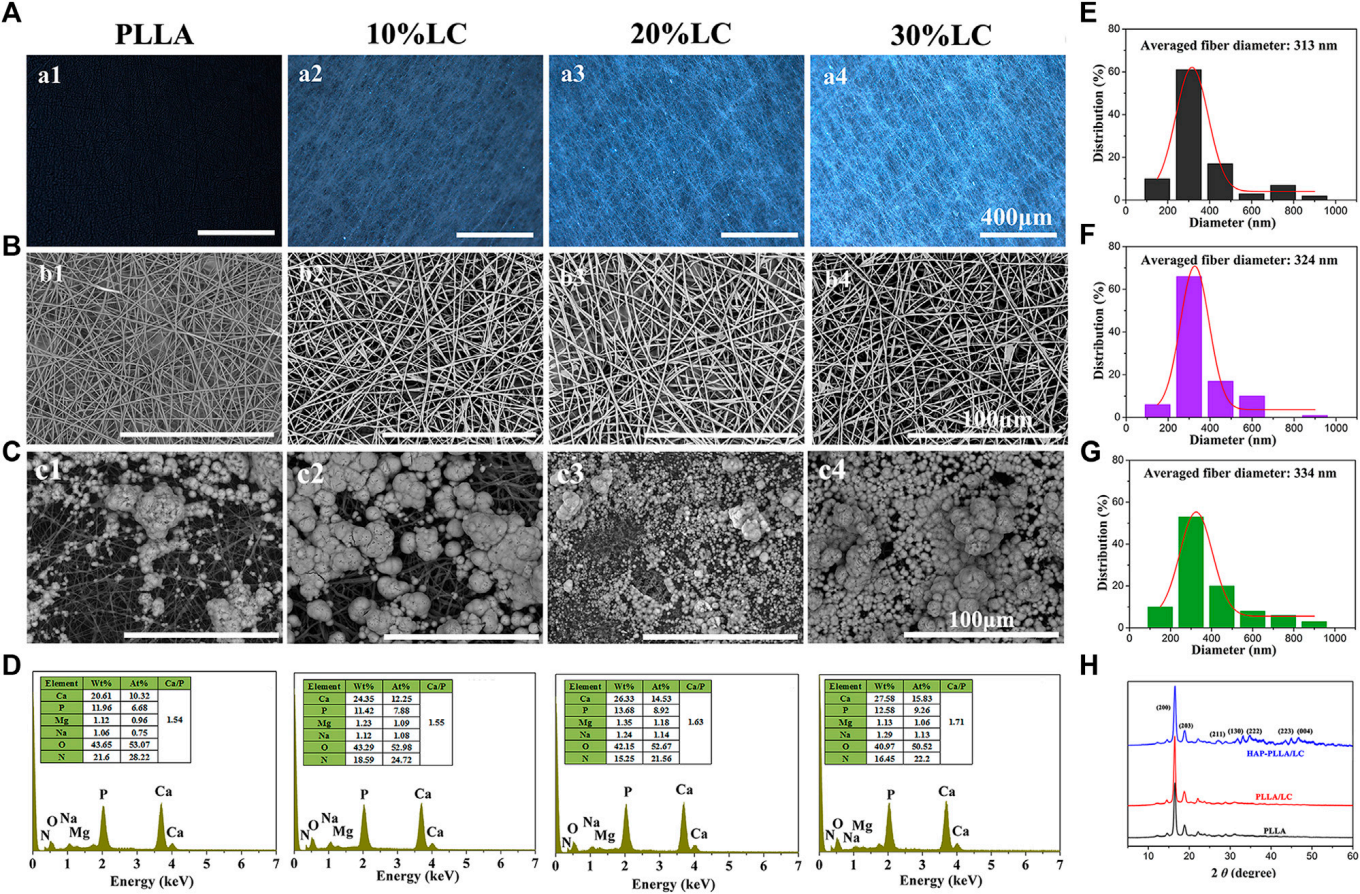
The polarizing optical microscopy **(**POM) picture **(A)** and scanning electron microscope (SEM) topography **(B)** of the PLLA composite scaffold with different LC contents; **(C)** the response to SEM morphologies and **(D)** energy dispersive spectrometer (EDS) spectra of poly (L-lactide) (PLLA) and PLLA/LC composite fibers after mineralized in 1.5× simulated body fluid (SBF) solution for 7 days. The fiber diameter distribution of **(E)** PLLA, **(F)** PLLA/LC, and **(G)** hydroxyapatite (HAP)-PLLA/LC. **(H)** X-ray diffraction patterns of three group samples.

The polarized images of the composites are shown in Figure 3. The PLLA matrix is black under polarized light, and the bright spot in the composite spinning material corresponds to the LC phase. As the LC content increased, the LC phase domains gradually increased, and the field of view under POM gradually became brighter. When the LC content was 30%, the material as a whole appeared bright. The LC phase exists in the spinning material in a special dispersed phase, and the LC texture is destroyed due to the presence of the PLLA polymer, which prevents the beautiful LC texture from being produced.

In order to investigate the effect of introduction of LC into the PLLA matrix on mineralization capacity, PLLA composites containing different LC contents were fabricated. It can be seen from SEM that both PLLA and composite materials exhibit comparable fiber specifications, indicating that LC introduced to a polymer system could provide strong interfacial interaction between inorganic and organic phase via silicon bonds to calcium ions, and therapy effectively induced HAP formation *in situ* on the material surface resulting in better mineralization capacity. In addition, according to the POM image, the fibers of the composite material have a root-like brightness rather than a block-like spot.

After mineralization of the SBF solution for 7 days, the minerals on the PLLA and PLLA/LC fiber membranes increased with increasing LC content, and the 20 and 30% PLLA/LC composite groups produced continuous and dense near-spherical apatite minerals; however, the formation of minerals on the PLLA and 10% PLLA/LC materials was relatively minimal. This is because pure PLLA has not been modified, and the surface of the fiber is smooth and flat with few reactive functional groups, which cannot provide an effective nucleation site for mineralization. After the addition of LC, the PLLA/LC composite material had a large number of LC domains, providing more nucleation sites on the fibers to promote nucleation and form more calcium phosphate minerals, which can be further converted into HAP and achieved favorable compatibility between inorganics and the polymer matrix. The mineralized product was formed by the accumulation of a large number of microspheres, so the minerals on the PLLA and PLLA/LC composite fibers shown in Figure 3C appeared nonuniform. However, with the increase in the LC composition, the uniformity of HA generated by the scaffold is higher, and almost spread across the entire membrane surface when the LC contents increased to 30%. The mineralized material was evenly spread over the surface of the material. The mineral morphology was the same as that obtained by Kokubo (Kokubo and Takadama, 2006; Nešović et al., 2018), where similar spherical morphology is observed as well, so it can be preliminarily determined that the formed near-spherical apatite minerals are HAP mineral.

To determine the mineralization of the PLLA and PLLA/LC that was mineralized for 7 days, the morphology was observed by SEM. The elemental composition of the minerals was analyzed by an EDS spectrometer attached to the SEM, and the C element was used as the reference. The elemental results are shown in Figure 3. After 7 days of mineralization, the ratio of calcium to phosphorus in the mineralized PLLA was 1.54, which is consistent with the difference in the morphology due to mineralization indicated by the ratios of 1.63 and 1.71 in the 20 and 30% mineralized PLLA/LC, further proving that the mineralization effect of the PLLA fiber scaffold surface is reduced compared with that of PLLA/LC. It is known that the minerals of the fiber material also contain Mg and Na, indicating that Mg^2+^ and Na^+^ are also involved in mineralization during the mineralization process and result in the formation of HAP that is not completely pure; however, small amounts of Mg promote bone formation and are also essential for the human body, so Mg is not a harmful impurity for bone repair materials. The mineralized 30% PLLA/LC component has the highest calcium–phosphorus ratio, which is close to the ratio of 1.67 of HAP. This further demonstrates that the minerals of the 30% PLLA/LC group better absorb Ca^2+^ and PO_4_^3+^ in solution and gradually grow and transform into HAP. At the same time, new nucleation sites can be formed on the minerals to facilitate nucleation and form more calcium phosphate minerals that can be further converted into HAP. Thus, the mineralized 30% PLLA/LC scaffolds with the highest mineralization capacity was chosen as an optimal group for following experiments.

In addition, the homologous fiber diameter distributions of electrospun PLLA, PLLA/LC, and HAP-PLLA/LC nanofibers were determined by typical SEM images (Figures 3E–G). The continuous and lapped morphology could be observed and their average diameters were about 313, 324, and 334 nm, respectively. These electrospinning nanofibers can effectively mimic the structure of ECM, which was beneficial to cell growth and proliferation (Li et al., 2019). XRD investigation was illustrated in Figure 3H; two sharp characteristic diffraction peaks were observed in 16.5° and 19.7°, which attributed to (200) and (203) planes of crystalline PLLA (Hu et al., 2017). There were no new characteristic peaks that appeared on PLLA/LC fiber because LC is an amorphous substance, whereas the HAP-PLLA/LC specimens showed typical characteristic peaks at 24.3°, 28.0°, 34.1°, 36.9°, 43.7°, and 48.2°, which correspond to the (002), (211), (130), (222), (213), and (004) crystal planes of HAP, respectively (Liu et al., 2018a). It was further verified that the generated minerals via biomimetic mineralization was HAP.

### Pore Size, Tensile Properties, Hydrophilicity, and *in vitro* Degradation of the Composites

**FIGURE 4:**
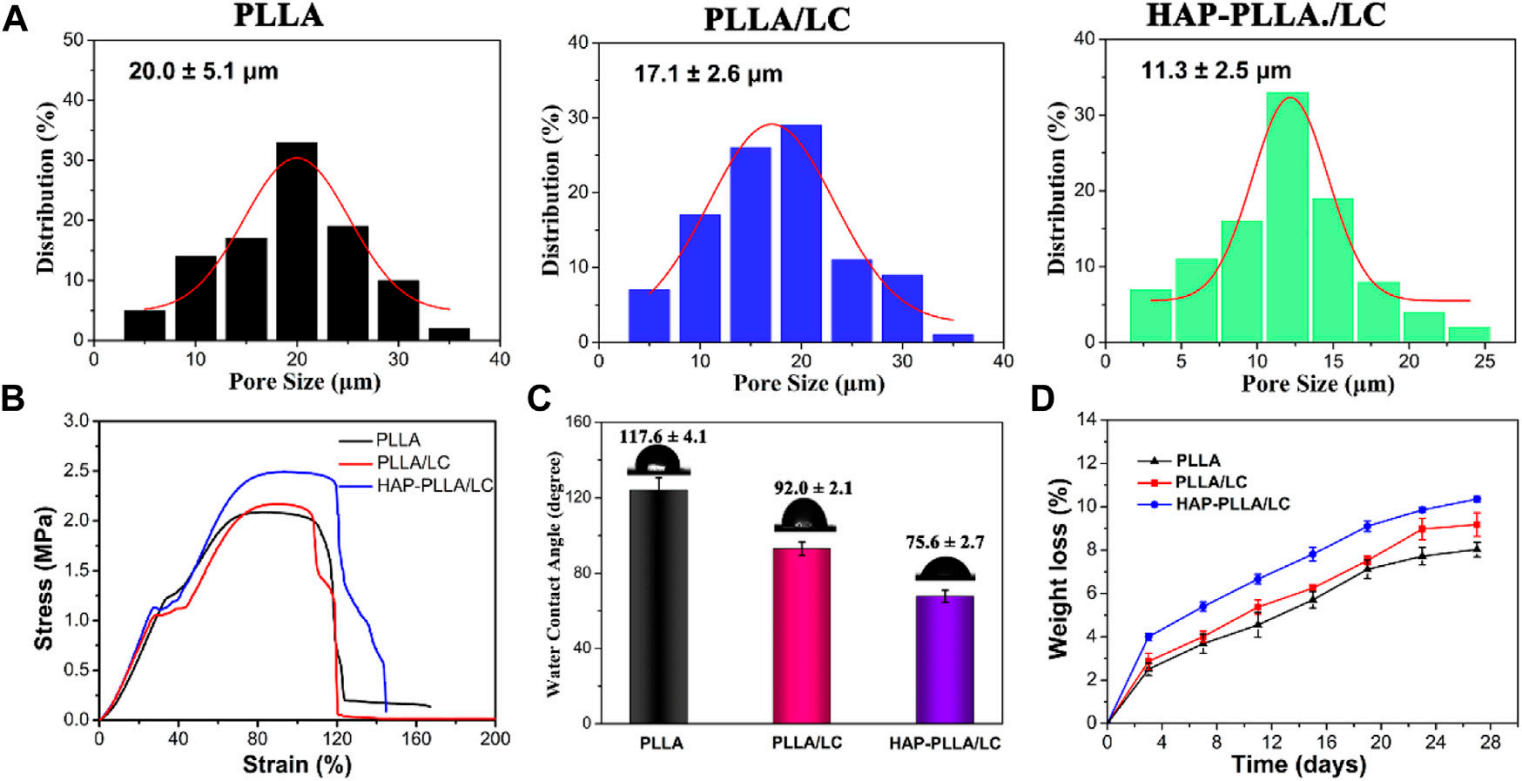
**(A)** Pore size of the resulting PLLA, PLLA/LC, and HAP-PLLA/LC fibers. **(B)** The representative stress–strain curves of resulting fibrous membranes, and **(C)** the corresponding water contact angle (CA) values of three group samples. **(D)** *In vitro* degradation curves of PLLA, PLLA/LC, and HAP-PLLA/LC scaffolds in SBF medium.

Figure 4A shows the pore size of porous fiber scaffolds. In comparison with the PLLA and PLLA/LC scaffold, the mineralized PLLA/LC specimens exhibit a decrease in the pore size. This may be explained that the formation of minerals depositing in the surface of fiber membrane, resulted in a decrease in the porosity of the *in situ* nanocomposite scaffolds. Similar phenomena have been reported in the literature (Chen et al., 2019c). Mechanical results showed that the PLLA/LC fibrous membrane displayed enhanced tensile properties after surface mineralization with HAP, leading to the stronger and stiffer HAP-PLLA/LC fibrous membranes (Figure 4B). Due to the inherent hydrophobicity of PLLA, the WCA of neat PLLA sample was measured to be 117.6°. After the LC was incorporated to the substrate, the WCA value of the PLLA/LC membrane was significantly decreased owing to the soft segment tetraethylene glycol in the hydrophilic LC (Figure 4C). Also, the formed HAP contributes to a great improvement in the hydrophilicity of fibrous membranes, which is attributed to the presence of hydroxyl groups in HAP (Shan et al., 2019). In addition, *in vitro* degradation of fibrous scaffolds in the SBF solution was evaluated by mass loss in 28 days. Compared with other groups, HAP-PLLA/LC exhibited the highest degradation rate. Obviously, incorporation of LC and HAP component into the polymer matrix could improve its hydrophilicity and disturb the structure of PLLA, thereby leading to the decrease of crystallinity and the acceleration of degradation process.

### Cellular Behaviors on the Surface of the Composites

**FIGURE 5:**
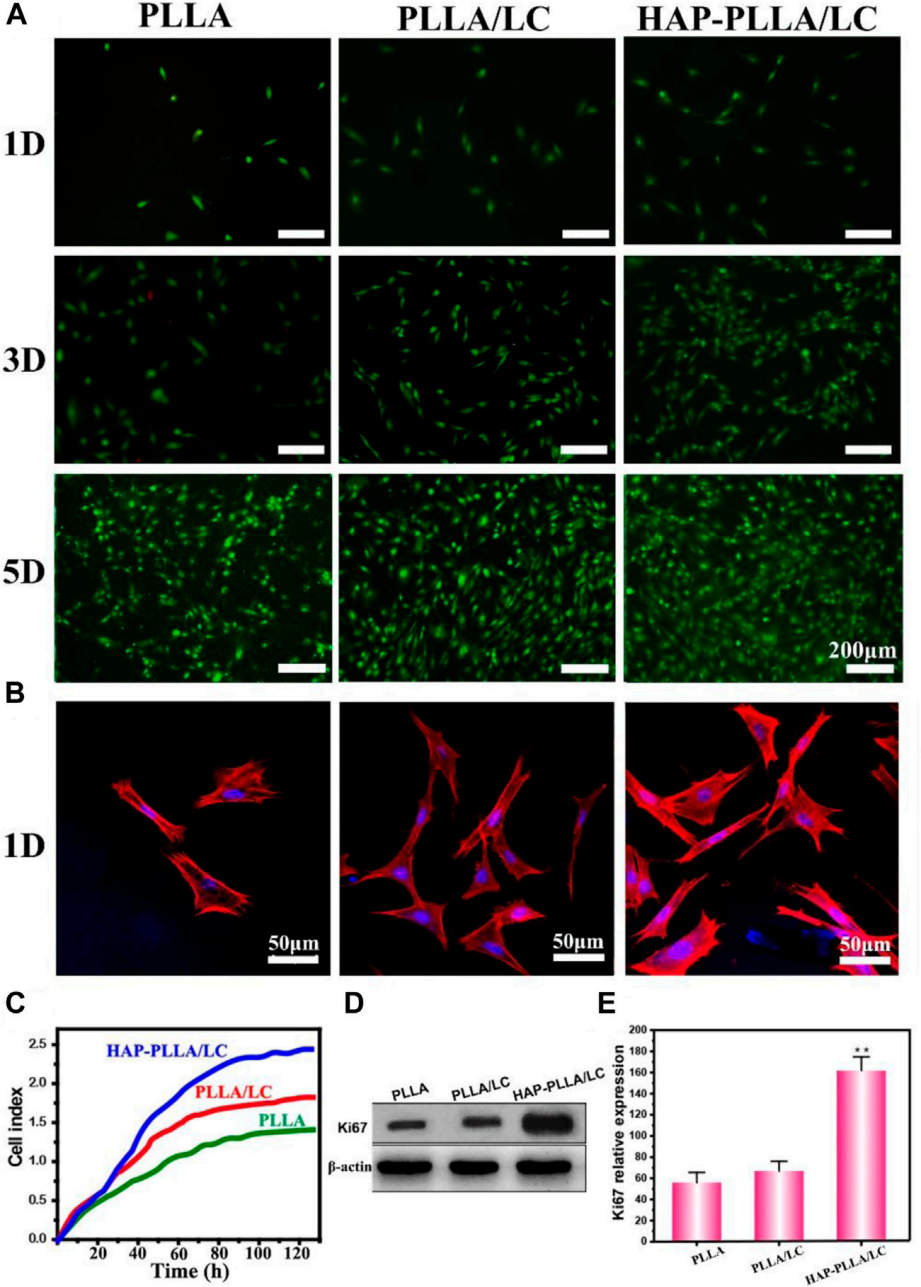
**(A)** Images of live/dead fluorescent staining of BMSCs cells on the PLLA, PLLA/LC, and HAP-PLLA/LC composites after culturing for 1, 3, and 5 days. **(B)** CLSM micrographs of BMSCs stained with rhodamine-conjugated phalloidin (red) and 4, 6-diamidino-2-phenylindole (DAPI) (blue) were cultured on the PLLA, PLLA/LC, and HAP- PLLA/LC composites for 1 day. **(C)** The cell growth curve of BMSCs. **(D,E)** The results of Ki67 protein expression on day 5 (*p < 0.05 and **p < 0.01).

The effects of the surfaces of materials of different compositions on the proliferation of BMSC cells on days 1, 3, and 5 are shown in Figure 5A. The results of the AO/EB staining showed no significant difference in the results for all groups on day 1. Moreover, the cells cultured in all groups proliferated significantly on days 3 and 5, but both the number of cells on the PLLA/LC and HAP-PLLA/LC groups was relatively greater than the number of cells in the PLLA group; especially, the HAP-PLLA/LC component was the largest, which is consistent with the results in the related literature (Sharma et al., 2015). When the cholesteryl ester LC contacts the cells, it can trigger the secretion of proteins by the cells, thereby promoting the adhesion and proliferation of the cells on the material. In agreement with the results generated from cell growth curves (Figure 5C), the HAP-PLLA/LC group exhibited better proliferation capacity than the other two groups. It can be attributed to polymer LCs exhibiting better fluidity that are closer to LC substances in living organisms, like proteins, nucleic acids, polysaccharides, and lipids, increasing the affinity of cells to make them more proliferative, as well as the cholesteric LC-incorporated improving the hydrophilicity and cell affinity leading to better cell proliferation results of the composite system (Hwang et al., 2002; Bera et al., 2015). The expression levels of the Ki67 protein (a typical proliferation marker) also confirmed this conclusion, which is notably increased in biomineralized PLLA/LC fiber compared with PLLA and PLLA/LC (Figures 5D,E). Taken together, these results suggest that HAP-PLLA/LC composite scaffold provided favorable condition for cell proliferation.

To better understand the distribution of cells on the material, the morphology and adhesion of BMSC cells cultured on the prepared material for 1 day were examined by LSCM (Figure 5B). Cells cultured on the PLLA/LC and HAP-PLLA/LC materials exhibited relatively good adhesion, while cells cultured on the PLLA materials showed little diffusion and exhibited only relatively weak adhesion. For the PLLA modified with LC, the pseudopods of the adherent cells were further stretched, and the diffusion area increased owing to LC-containing scaffold, which provides a suitable substrate to support three-dimensional (3D) cell growth *via* stimulation of anisotropic viscoelastic and biochemical interactions between the receptor and ligand to co-regulate the cell–matrix relationship (Hwang et al., 2002), while the adherent cells cultured on the mineralized HAP-PLLA/LC material showed the largest spreading area. It is obvious that the number of cells distributed on the LC-containing material was large, and these cells showed mostly an elongated polygon shape and were evenly distributed on the material, indicating that they were more likely to grow and migrate on the above material.

Since the LC has certain fluidity, it contributes to an improvement in the cell affinity and biocompatibility of the film. Furthermore, the presence of cholesterol ester groups facilitates sensitivity to cell shrinkage, promotes the secretion of adhesion proteins by cells, enhances the physical properties of cells during attachment and proliferation, and promotes cell adhesion and proliferation (Soon et al., 2009; Soon et al., 2014).

**FIGURE 6:**
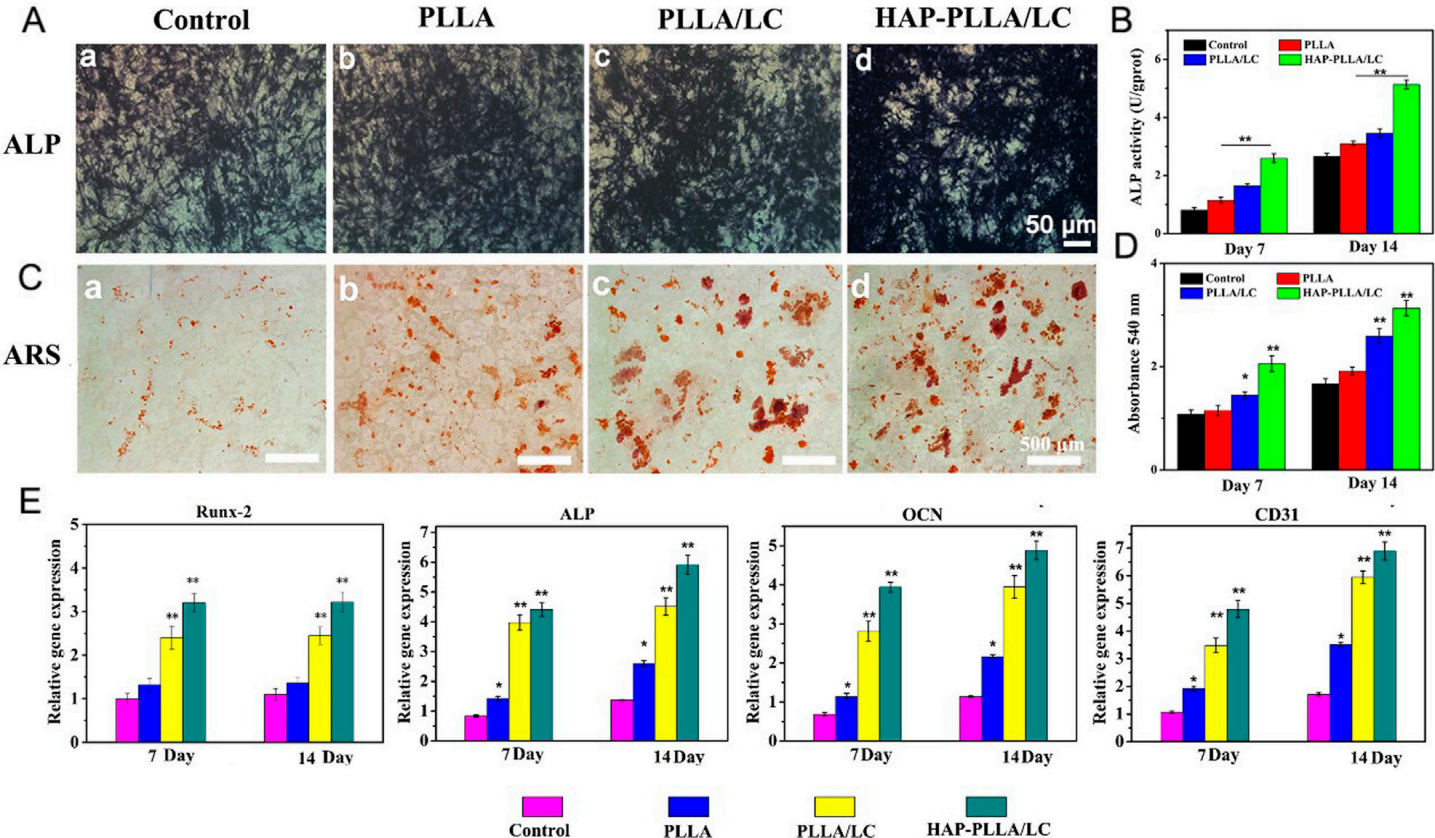
**(A)** Alkalinephosphatase (ALP) staining and **(B)** its quantitative analysis of BMSC cells cultured on the scaffolds for 14 days. **(C)** Alizarin red (AR) staining and **(D)** its quantitative analysis of BMSC cells cultured on a culture plate, PLLA, PLLA/LC, and HAP-PLLA/LC fiber scaffold for 14 days, respectively. **(E)** Real-time quantitative PCR (RT-qPCR) analysis of osteogenic and vascularized-related gene expression of cells after culturing for 7 and 14 days.

To assess the early effect of LC materials on the osteogenic activity of cells, ALP staining and the ALP activity were examined for qualitative and quantitative analyses. It can be found that PLLA/LC and HAP-PLLA/LC show more positive gray-black particles than the other two groups, and its quantitative analysis also indicated that the presence of LC caused the increase in the ALP activity in a dose- and time-dependent manner (Figures 6A,B). Similarly, the AR staining study showed remarkably increased calcium deposition, with many bulky calcium nodules in plump shapes shown in the HAP-PLLA/LC group (Figures 6C,D). It was reported that the Si element plays a critical role in promoting osteogenesis (Zhang et al., 2019; Tamburaci and Tihminlioglu, 2020), and the formation of HAP can also significantly increase the ability to promote osteogenic differentiation, leading to improvement of the osteogenic activity (Saffarian Tousi et al., 2013).

The results of RT-qPCR show that different materials have different effects on gene expression. Osteoblasts differentiate into mature cells through three stages: cell proliferation, ECM maturation, and matrix mineralization. Osteoblasts begin to differentiate and produce ECM after cell proliferation to form bone tissue once the matrix matures (López-Alvarez et al., 2009). It is well known that the initiation and termination of differentiation is necessarily accompanied by the expression of a series of intracellularly modified genes (Liu et al., 2018b). Overall, on days 7 and 14, the expression of osteogenic genes showed no significant enhancement in cells on the PLLA scaffold compared with that in the control group. However, on the LC-modified and mineralized scaffolds, particularly on the HAP-PLLA/LC scaffold, the enhancement was significant. In addition, gene expression on the HAP-PLLA/LC scaffold was higher than that on the PLLA/LC scaffold, indicating that the mineralized material has better osteogenic function.

Runx-2 is a key transcription factor for osteoblast differentiation that directly regulates extracellular matrix proteins (Zhang et al., 2011; Shackleton et al., 2006). It is also the primary transcriptional element that initiates the transcriptional program of the osteogenic lineage, thereby upregulating other downstream genes associated with bone (Kim et al., 2012). Runx-2 is a very early marker of osteogenic induction, and its upregulation usually only persists for the first 3 days. As shown in Figure 6E, the expression of Runx-2 gene does not increase with the incubation time. The increase in Runx-2 gene expression was greater on the HAP-PLLA/LC scaffold than on the PLLA and PLLA/LC scaffolds. This indicates that the LC composite material after mineralization has a better effect than the single component, and the mineralized substance has a better effect on osteogenesis in the early stage of differentiation than PLLA/LC. ALP reflects the degree of differentiation of osteoblasts and its activity will peak after ECM maturation (Bonnelye et al., 2008; Heo et al., 2014). Compared with that in the control group, the LC-modified scaffold significantly increased the expression of ALP (**p < 0.01). For the HAP-PLLA/LC and PLLA/LC scaffolds, a gradual increase in the expression of the Runx-2 and ALP genes after mineralization can enhance the maturation of the ECM and increase the mineralization efficiency. The OCN gene is a marker of late osteoblast differentiation (Lin et al., 2009) and affects the maturation and mineralization of osteoblasts (Radice et al., 2007). The detectable difference in the expression of the OCN gene in the PLLA scaffold was not obvious, but the expression of the OCN gene in the PLLA/LC and HAP-PLLA/LC groups showed significant increase compared with control group, and there was significant differential expression (**p < 0.01). The expression of OCN in all groups was closely related to the expression changes of the other two genes (Runx-2 and ALP). As a result of the increase in the expression of the OCN gene, BMSC cells transformed into mature bone cells and entered the mineralization phase.

At the same time, we also determined the expression of the vascularization factor CD31, which is a platelet–endothelial cell adhesion molecule that promotes angiogenesis and granulation tissue formation, thereby enhancing epithelial regeneration (Xu and Huang, 2007). It is commonly used to assess endothelial cell angiogenic capacity (Na et al., 2016). Angiogenic gene expression in the control and PLLA groups was lower than that in the PLLA/LC and HAP-PLLA/LC scaffold groups, and the expression on the LC-modified scaffold was good and even improved. The expression of these genes was further increased after incubation of cells with the mineralized scaffold. Interestingly, the mineralized material not only shows good osteogenic activity but also exhibits considerable angiogenic activity in the presence of the same LC content designed in this study. This may be attributed to the LC-enriched fibrous scaffold that mimics the microenvironment for vascularization due to better fluidity that are closer to LC substances in living organisms, like proteins, nucleic acids, polysaccharides, and lipids (Neffe et al., 2015; Huang et al., 2019b; Xie et al., 2020), which revealed the possibility of stimulators to the regulation of the angiogenic capacity by LC with anisotropic viscoelastic matrix.

Taken together, HAP-PLLA/LC composite scaffold displayed good osteogenic and vascular performance *in vitro.* For mineralized PLLA/LC system, LC was introduced to a polymer system that could provide strong interfacial interaction between inorganic and organic phase *via* silicon bonds to calcium ions. Therapy effectively induced HAP formation *in situ* on the material surface resulting in better mineralization capacity. On the one hand, the presence of Si component in LC may affect the osteogenic differentiation and angiogenic ability of BMSCs cells (Zhang et al., 2020; Zhang et al., 2021). On the other hand, the HA mineral, as a bone-like mineral, has good osteoconductive activity. Obviously, the LC incorporation and HA immobilization played a synergistic effect on promoting the expression of the related osteogenic genes. Furthermore, the results of PCR showed that the LC-containing group could upregulate the expression of the angiogenic CD31 gene. This may be related to Si, which is a crucial element for the induction of angiogenesis *via* activation of HIF-1α, more importantly because the rough surface nanotopography and anisotropic viscoelastic property of the composite nanomaterial provides a mimic of the microenvironment of vascularization, thus, directly and indirectly promoting the angiogenic activity (Yu et al., 2017; Wei et al., 2020). Angiogenesis is an indispensable guarantee for favorable functions of osteoblasts and rapid generation of bone tissue, which further promotes the osteogenic activity. Therefore, our research has successfully demonstrated that the HAP-PLLA/LC scaffold is anticipated to be applied as a hopeful bone regeneration material with improved osteogenesis and angiogenesis.

### Histomorphometric Analyses

**FIGURE 7:**
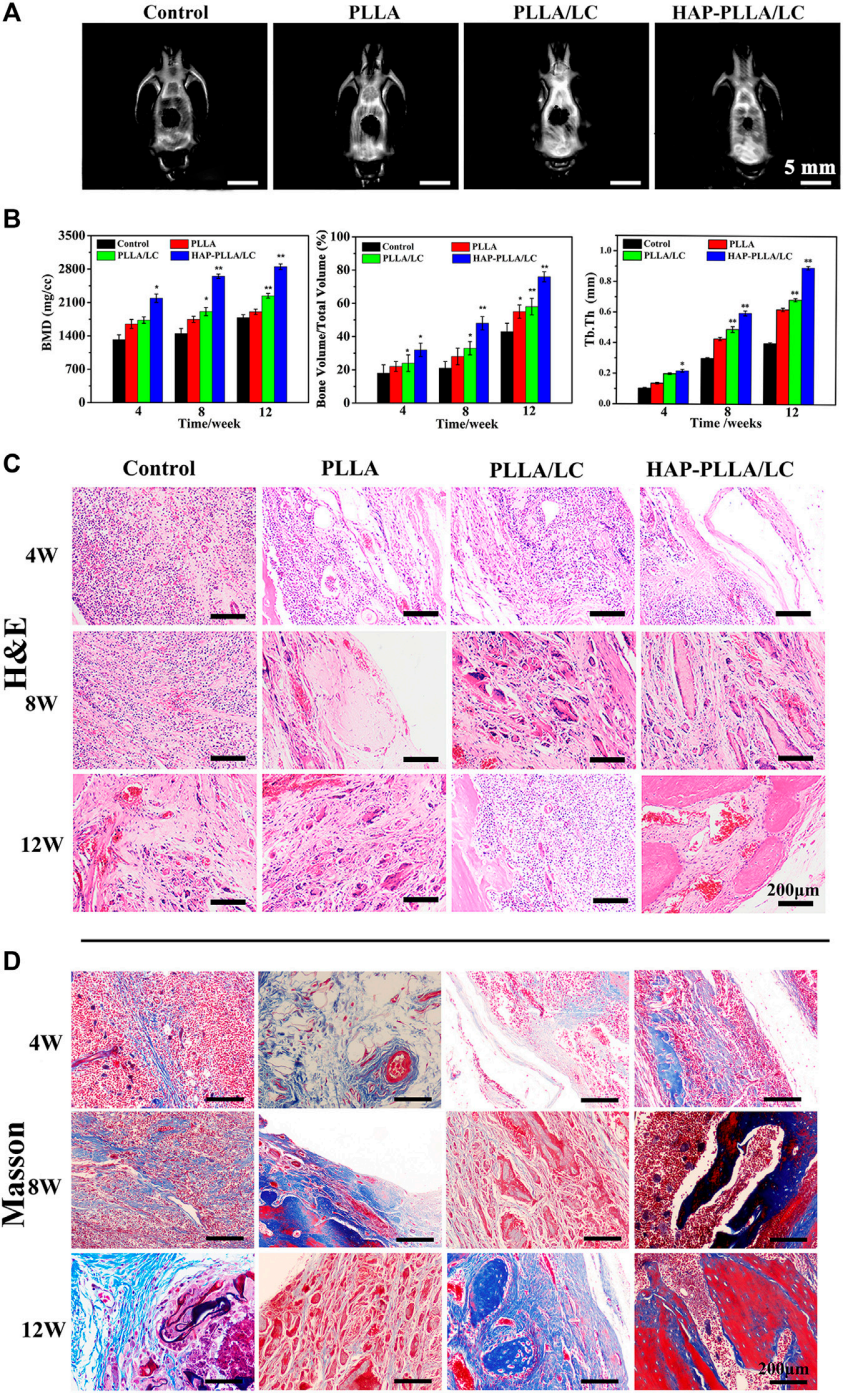
**(A)** Micro-CT results of bone defect of 12 weeks after surgery; **(B)** quantitative analysis of bone mineral density (BMD), the bone volume/total volume fraction (BV/TV), and trabecular thickness (Tb.Th). **(C)** Hematoxylin and eosin (H&E) staining and **(D)** Masson’s staining of samples (including control, PLLA, PLLA/LC, and HAP-PLLA/LC filled areas at 4, 8, and 12 weeks of postimplantation.

The H&E (Figure 7C) and Masson staining (Figure7D) results showed the poor recovery of the bone defects in the control group, where there were few new collagen fibers, and the bone calcium content was low. After the implantation of the PLLA/LC and HAP-PLLA/LC materials, the bone remodeling process was significantly improved, and the bone defects showed signs of recovery. After 8 weeks, there was a small amount of undegraded material in the PLLA material, and a large number of osteoblasts appeared in both the PLLA/LC and HAP-PLLA/LC materials, and both began to show new bone formation. After 12 weeks of material implantation, the BMDs of control, PLLA, PLLA/LC, and HAP-PLLA/LC groups are 1,710 ± 153, 1,869 ± 135, 2,409 ± 141, and 3,117 ± 169 mg/cm^3^, respectively, and the corresponding BV/TV ratios are 38.6, 54.9, 58.3, and 76.0%, respectively (Figure 7B). Obviously, the new bone volume was increased, especially in the HAP-PLLA/LC material, showing the highest bone density and the highest Tb.Th value (0.81 ± 0.17 mm), which is consistent with the Micro-CT results (Figure 7A), demonstrating that the bone regeneration could be further promoted by HAP-PLLA/LC scaffold. In addition, the entire dynamic process of defect repair was observed in the bone at all time points. In the Masson’s trichrome staining results (Figure 7D), the collagen fibers were dyed blue; the change in process first showed the formation of collagen into strips, and then bundles, and then finally into pieces, which dynamically reflected the process of new bone formation. Apparently, the new bone coverage area in the PLLA/LC and HAP-PLLA/LC materials was significantly greater than that in the A groups at 8 and 12 weeks. The rate of collagen fiber synthesis and the bone calcium content increased in combination with an increase in the treatment time. The new bone grew from the peripheral part of the defect into the central part. It was not difficult to come to the conclusion that the addition of LC and nHAP showed a significant advantage compared with that of pure PLLA in the regeneration of bone defects. It is probable that the HAP-PLLA/LC material provides a more stable local environment than the PLLA and PLLA/LC materials, contributing to better bone tissue regeneration.

### Immunohistochemical Examination of BMP-2, Osteocalcin, and CD31

To provide further verification of the bioactivity of the prepared fiber scaffold, immunohistochemical examinations of BMP-2, OCN, and CD31 were performed to evaluate the material implanted in the different groups. *Via* immunohistochemistry, bone formation was further confirmed by BMP-2 and OCN staining. Both BMP-2 (Figure 7A) and OCN (Figure 7B) staining produced intense brown stains for both time periods and showed greatly enhanced spreading at 8 weeks. The results obtained for OCN immunohistochemical staining revealed moderate expression of OCN at the edges of the host bone in the blank control group, while OCN expressed more strongly in the PLLA/LC and HAP-PLLA/LC group and, therefore, exhibited better bone regeneration in the composite scaffold group. After 12 weeks of treatment, the OCN expression showed a recession owing to newly generated bone in PLLA/LC and HAP-PLLA/LC group. In short, BMP-2 (component of osteoid) and the ground substance OCN were identified by sequential section staining, and more mature bone could also be observed in the detailed staining results of HAP-PLLA/LC, which confirms the high capacity of bone repair. The above results explain the effects of PLLA/LC and HAP-PLLA/LC materials on the differentiation of hMSCs into the bone. Taken together, the results show that HAP-PLLA/LC is a potential candidate for bone repair and regeneration processes *in vivo*.

**FIGURE 8:**
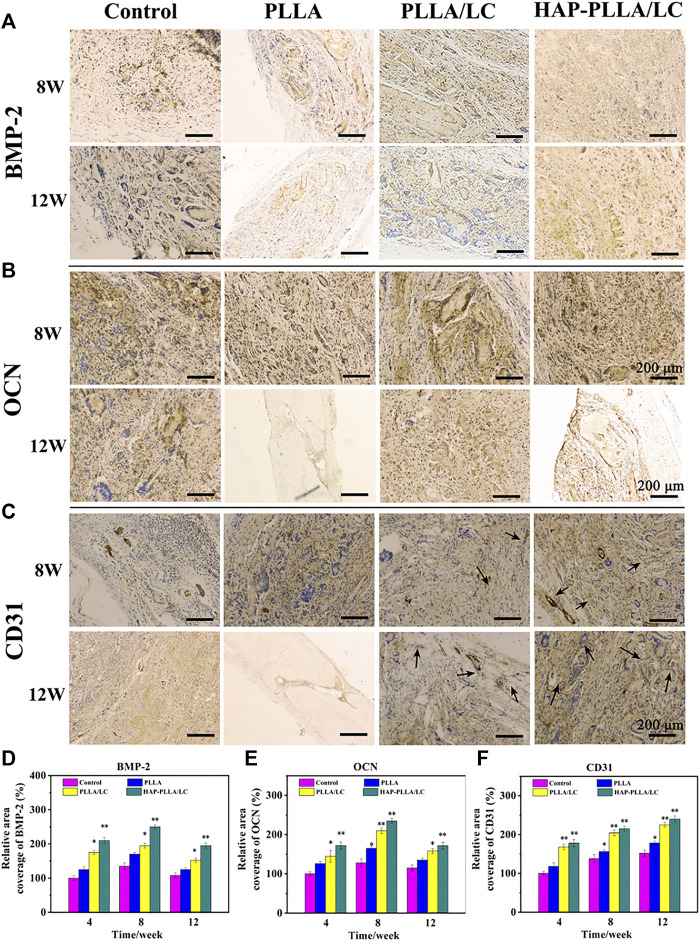
The immunohistochemical BMP-2, osteocalcin (OCN), and CD31 examinations of the osteogenic potential of control, PLLA, PLLA/LC, and HAP-PLLA/LC, respectively, were performed at 4, 8, and 12 weeks. The quantified analysis of **(D)** BMP-2, **(E)** OCN, and **(F)** CD31 at 4, 8, and 12 weeks after surgery.

CD31 is a cluster of differentiation molecule recognized as a marker of new endothelial cells and is commonly used for neonatal microvessel counting to assess the angiogenesis of implanted material (Ferrè et al., 2010). Hence, immunohistochemical staining of CD31 was conducted as shown in Figure 7C. The expression level of CD31, a marker of neovascularization, illustrated a larger number of newly formed vessels in the PLLA/LC and HAP-PLLA/LC groups compared with that in the control and PLLA groups. The highest density of neovasculature appeared in the HAP-PLLA/LC group at 8 weeks, which indicated that the positive signal became more intense at 8 weeks to indicate complete new bone formation. With the prolongation of time, the degree of neovascularization in the PLLA/LC and HAP-PLLA/LC groups decreased compared with that in the control group. Correspondingly, the quantitative analysis of newly formed vessels and total vessels (Figure 8F) also revealed that the density and number of positive vessels were remarkably increased by the incorporation of LC and HAP into the PLLA system. Such a phenomenon could be attributed to the occurrence of peak angiogenesis at 8 weeks postoperatively. The scaffold with the nHAP component shows the capacity to stimulate gene expression, accelerate the differentiation of osteoblasts, and promote the secretion of growth factors and the generation of ECM (Lai et al., 2015). At the same time, the relative quantitative results of BMP-2, OCN, and CD31 at 4, 8, and 12 weeks showed that the expression level in the HAP-PLLA/LC was higher than other groups, as shown in Figures 7D–F. In addition, it was reported that LC can also promote the secretion of vascular-related cytokines and promote the adhesion and proliferation of vascular endothelial cells and the formation of capillaries. These functions lay a good foundation for the regeneration of the periosteum to achieve the goal of accelerated bone regeneration.

## Conclusion

In this work, fiber composite material with an LC state similar to that in human tissue was successfully prepared by electrospinning process. The PLLA composite material with the LC content of 30% exhibited a superior LC state. The PLLA/LC composite has good mineralization ability and forms continuous hydroxyapatite. The cell experiment shows that the prepared composites could significantly promote BMSC attachment, proliferation, as well as osteogenic-related and angiogenic-related gene expression of MC3T3-E1 cells, thereby significantly facilitating the osteogenic differentiation and angiogenic activity. The *in vivo* results demonstrated that LC-modified PLLA and biomineralized inorganic–organic hybrid HAP-PLLA/LC composite fibers have enhanced osteoconductivity and osteogenesis potential and can also accelerate bone formation in preclinical animal models. All of these data indicated that the mineralized PLLA/LC composite scaffolds may be expected to possess potential biomedical applications in bone tissue engineering.

## Data Availability

The original contributions presented in the study are included in the article/Supplementary Material, further inquiries can be directed to the corresponding authors.

## References

[B1] Barnes C. P. Sell S. A. Boland E. D. Simpson D. G. Bowlin G. L. (2007). Nanofiber Technology: Designing the Next Generation of Tissue Engineering Scaffolds. Adv. Drug Deliv. Rev. 59, 1413–1433. 10.1016/j.addr.2007.04.022 17916396

[B2] Bera T. Freeman E. J. McDonough J. A. Clements R. J. Aladlaan A. Miller D. W. (2015). Liquid Crystal Elastomer Microspheres as Three-Dimensional Cell Scaffolds Supporting the Attachment and Proliferation of Myoblasts. ACS Appl. Mater. Inter. 7, 14528–14535. 10.1021/acsami.5b04208 26075811

[B3] Bonnelye E. Chabadel A. Saltel F. Jurdic P. (2008). Dual Effect of Strontium Ranelate: Stimulation of Osteoblast Differentiation and Inhibition of Osteoclast Formation and Resorption *In Vitro* . Bone 42, 129–138. 10.1016/j.bone.2007.08.043 17945546

[B4] Charvolin J. Sadoc J. F. (2012). About Collagen, a Tribute to Yves Bouligand. Interf. Focus 2, 567–574. 10.1098/rsfs.2012.0014 PMC3438575 24098840

[B5] Chen S. Zhu L. Wen W. Lu L. Zhou C. Luo B. (2019). Fabrication and Evaluation of 3D Printed Poly(L-Lactide) Scaffold Functionalized with Quercetin-Polydopamine for Bone Tissue Engineering. Acs Biomater. Sci. Eng. 5, 2506–2518. 10.1021/acsbiomaterials.9b00254 33405757

[B6] Chen X. Zhu L. Liu H. Wen W. Li H. Zhou C. (2019). Biomineralization Guided by Polydopamine-Modifed poly(L-Lactide) Fibrous Membrane for Promoted Osteoconductive Activity. Biomed. Mater. 14, 055005. 10.1088/1748-605X/ab2f2d 31271155

[B7] Chen X. Zhu L. Wen W. Lu L. Luo B. Zhou C. (2019). Biomimetic Mineralisation of Eggshell Membrane Featuring Natural Nanofiber Network Structure for Improving its Osteogenic Activity. Colloids Surf. B Biointerfaces 179, 299–308. 10.1016/j.colsurfb.2019.04.009 30981065

[B8] Ferrè S. Baldoli E. Leidi M. Maier J. A. (2010). Magnesium Deficiency Promotes a Pro-atherogenic Phenotype in Cultured Human Endothelial Cells via Activation of NFkB. Biochim. Biophys. Acta 1802, 952–958. 10.1016/j.bbadis.2010.06.016 20600865

[B9] Ha Y. Yang J. Tao F. Wu Q. Song Y. J. Wang H. R. (2018). Phase-Transited Lysozyme as a Universal Route to Bioactive Hydroxyapatite Crystalline Film. Adv. Funct. Mater. 28, 1704476. 10.1002/adfm.201704476

[B10] Hamley I. W. (2010). Liquid crystal Phase Formation by Biopolymers. Soft Matter 6, 1863–1871. 10.1039/b923942a

[B11] Heo D. N. Ko W. K. Bae M. S. Lee J. B. Lee D. W. Byun W. (2014). Enhanced Bone Regeneration with a Gold Nanoparticle-Hydrogel Complex. J. Mater. Chem. B 2, 1584–1593. 10.1039/c3tb21246g 32261377

[B12] Hu C. Li Z. Wang Y. Gao J. Dai K. Zheng G. (2017). Comparative Assessment of the Strain-Sensing Behaviors of Polylactic Acid Nanocomposites: Reduced Graphene Oxide or Carbon Nanotubes. J. Mater. Chem. C 5, 2318–2328. 10.1039/c6tc05261d

[B13] Hu D. Cui Y. Mo K. Wang J. Huang Y. Miao X. (2020). Ultrahigh Strength Nanocomposite Hydrogels Designed by Locking Oriented Tunicate Cellulose Nanocrystals in Polymeric Networks. Composites B: Eng. 197, 108118. 10.1016/j.compositesb.2020.108118

[B14] Huang L. Tan J. Li W. Zhou L. Liu Z. Luo B. (2019). Functional Polyhedral Oligomeric Silsesquioxane Reinforced Poly(lactic Acid) Nanocomposites for Biomedical Applications. J. Mech. Behav. Biomed. Mater. 90, 604–614. 10.1016/j.jmbbm.2018.11.002 30500698

[B15] Huang L. Zhu Z. Wu D. Gan W. Zhu S. Li W. (2019). Antibacterial Poly (Ethylene Glycol) Diacrylate/chitosan Hydrogels Enhance Mechanical Adhesiveness and Promote Skin Regeneration. Carbohydr. Polym. 225, 115110. 10.1016/j.carbpol.2019.115110 31521272

[B16] Hwang J. J. Iyer S. N. Li L. S. Claussen R. Harrington D. A. Stupp S. I. (2002). Self-assembling Biomaterials: Liquid crystal Phases of Cholesteryl oligo(L-Lactic Acid) and Their Interactions with Cells. Proc. Natl. Acad. Sci. U S A. 99, 9662–9667. 10.1073/pnas.152667399 12119419 PMC124968

[B17] Jewell S. A. (2011). Living Systems and Liquid Crystals. Liquid Crystals 38, 1699–1714. 10.1080/02678292.2011.603846

[B18] Kim H. K. Kim J. H. Park D. S. Park K. S. Kang S. S. Lee J. S. (2012). Osteogenesis Induced by a Bone Forming Peptide from the Prodomain Region of BMP-7. Biomaterials 33, 7057–7063. 10.1016/j.biomaterials.2012.06.036 22795855

[B19] Kokubo T. Takadama H. (2006). How Useful Is SBF in Predicting *In Vivo* Bone Bioactivity. Biomaterials 27, 2907–2915. 10.1016/j.biomaterials.2006.01.017 16448693

[B20] Lai G. J. Shalumon K. T. Chen J. P. (2015). Response of Human Mesenchymal Stem Cells to Intrafibrillar Nanohydroxyapatite Content and Extrafibrillar Nanohydroxyapatite in Biomimetic Chitosan/silk Fibroin/nanohydroxyapatite Nanofibrous Membrane Scaffolds. Int. J. Nanomedicine 10, 567–584. 10.2147/IJN.S73780 25609962 PMC4298333

[B21] Li W. Wu D. Zhu S. Liu Z. Luo B. Lu L. (2019). Sustained Release of Plasmid DNA from PLLA/POSS Nanofibers for Angiogenic Therapy. Chem. Eng. J. 365, 270–281. 10.1016/j.cej.2019.02.043

[B22] Lin L. Chow K. L. Leng Y. (2009). Study of Hydroxyapatite Osteoinductivity with an Osteogenic Differentiation of Mesenchymal Stem Cells. J. Biomed. Mater. Res. A. 89, 326–335. 10.1002/jbm.a.31994 18431794

[B23] Liu H. Wen W. Chen S. Zhou C. Luo B. (2018). Preparation of Icariin and Deferoxamine Functionalized Poly(l-Lactide)/chitosan Micro/Nanofibrous Membranes with Synergistic Enhanced Osteogenesis and Angiogenesis. ACS Appl. Bio Mater. 1, 389–402. 10.1021/acsabm.8b00129 35016399

[B24] Liu Z. Hu D. Huang L. Li W. Tian J. Lu L. (2018). Simultaneous Improvement in Toughness, Strength and Biocompatibility of Poly(lactic Acid) with Polyhedral Oligomeric Silsesquioxane. Chem. Eng. J. 346, 649–661. 10.1016/j.cej.2018.03.077

[B25] Lockwood N. A. Mohr J. C. Ji L. Murphy C. J. Palecek S. P. de Pablo J. J. (2006). Thermotropic Liquid Crystals as Substrates for Imaging the Reorganization of Matrigel by Human Embryonic Stem Cells. Adv. Funct. Mater. 16, 618–624. 10.1002/adfm.200500768

[B26] López-Alvarez M. Solla E. L. González P. Serra J. León B. Marques A. P. (2009). Silicon-hydroxyapatite Bioactive Coatings (Si-HA) from Diatomaceous Earth and Silica. Study of Adhesion and Proliferation of Osteoblast-like Cells. J. Mater. Sci. Mater. Med. 20, 1131–1136. 10.1007/s10856-008-3658-0 19089599

[B27] Mitov M. (2017). Cholesteric Liquid Crystals in Living Matter. Soft Matter 13, 4176–4209. 10.1039/c7sm00384f 28589190

[B28] Na W. Park J. W. An J. H. Jang J. (2016). Size-controllable Ultrathin Carboxylated Polypyrrole Nanotube Transducer for Extremely Sensitive 17β-Estradiol FET-type Biosensors. J. Mater. Chem. B 4, 5025–5034. 10.1039/c6tb00897f 32264029

[B29] Neffe A. T. Pierce B. F. Tronci G. Ma N. Pittermann E. Gebauer T. (2015). One Step Creation of Multifunctional 3D Architectured Hydrogels Inducing Bone Regeneration. Adv. Mater. 27, 1738–1744. 10.1002/adma.201404787 25601165

[B30] Nešović K. Janković A. Kojić V. Vukašinović-Sekulić M. Perić-Grujić A. Rhee K. Y. (2018). Silver/poly(vinyl Alcohol)/chitosan/graphene Hydrogels – Synthesis, Biological and Physicochemical Properties and Silver Release Kinetics. Composites Part B: Eng. 154, 175–185.

[B31] Palffy-Muhoray P. (2007). The Diverse World of Liquid Crystals. Phys. Today 60, 54–60. 10.1063/1.2784685

[B32] Radice S. Kern P. Bürki G. Michler J. Textor M. (2007). Electrophoretic Deposition of Zirconia-Bioglass Composite Coatings for Biomedical Implants. J. Biomed. Mater. Res. A. 82, 436–444. 10.1002/jbm.a.31162 17295244

[B33] Rey A. D. (2010). Liquid crystal Models of Biological Materials and Processes. Soft Matter 6, 3402–3429. 10.1039/b921576j

[B34] Saffarian Tousi N. Velten M. F. Bishop T. J. Leong K. K. Barkhordar N. S. Marshall G. W. (2013). Combinatorial Effect of Si4+, Ca2+, and Mg2+ Released from Bioactive Glasses on Osteoblast Osteocalcin Expression and Biomineralization. Mater. Sci. Eng. C Mater. Biol. Appl. 33, 2757–2765. 10.1016/j.msec.2013.02.044 23623093

[B35] Satiat-Jeunemaitre B. (1992). Spatial and Temporal Regulations in Helicoidal Extracellular Matrices: Comparison between Plant and Animal Systems. Tissue Cell 24, 315–334. 10.1016/0040-8166(92)90049-d 1636171

[B36] Shackleton M. Vaillant F. Simpson K. J. Stingl J. Smyth G. K. Asselin-Labat M. L. (2006). Generation of a Functional Mammary Gland from a Single Stem Cell. Nature 439, 84–88. 10.1038/nature04372 16397499

[B37] Shan L. Fan W. Wang W. Tang W. Yang Z. Wang Z. (2019). Organosilica-Based Hollow Mesoporous Bilirubin Nanoparticles for Antioxidation-Activated Self-Protection and Tumor-specific Deoxygenation-Driven Synergistic Therapy. ACS nano 13, 8903–8916. 10.1021/acsnano.9b02477 31374171

[B38] Sharma A. Neshat A. Mahnen C. J. Nielsen A. D. Snyder J. Stankovich T. L. (2015). Biocompatible, Biodegradable and Porous Liquid Crystal Elastomer Scaffolds for Spatial Cell Cultures. Macromol Biosci. 15, 200–214. 10.1002/mabi.201400325 25303674

[B39] Soon C. F. Omar W. I. Berends R. F. Nayan N. Basri H. Tee K. S. (2014). Biophysical Characteristics of Cells Cultured on Cholesteryl Ester Liquid Crystals. Micron 56, 73–79. 10.1016/j.micron.2013.10.011 24231674

[B40] Soon C. F. Youseffi M. Blagden N. Berends R. Lobo S. B. Javid F. A. (2009). Characterization and Biocompatibility Study of Nematic and Cholesteryl Liquid Crystals. Lect Notes Eng. Comp., 1872.

[B41] Tamburaci S. Tihminlioglu F. (2020). Chitosan-hybrid Poss Nanocomposites for Bone Regeneration: The Effect of Poss Nanocage on Surface, Morphology, Structure and *In Vitro* Bioactivity. Int. J. Biol. Macromol 142, 643–657. 10.1016/j.ijbiomac.2019.10.006 31622724

[B42] Wei Y. Shi M. Zhang J. Zhang X. Shen K. Wang R. (2020). Autologous Versatile Vesicles‐Incorporated Biomimetic Extracellular Matrix Induces Biomineralization. Adv. Funct. Mater. 30, 2000015. 10.1002/adfm.202000015

[B43] Xie W. Ouyang R. Wang H. Li N. Zhou C. (2020). Synthesis and Cytotoxicity of Novel Elastomers Based on Cholesteric Liquid Crystals. Liquid Crystals 47, 449–464. 10.1080/02678292.2019.1657594

[B44] Xu H. B. Huang Z. Q. (2007). Vasorelaxant Effects of Icariin on Isolated Canine Coronary Artery. J. Cardiovasc. Pharmacol. 49, 207–213. 10.1097/FJC.0b013e3180325abe 17438405

[B45] Yu Y. Jin G. Xue Y. Wang D. Liu X. Sun J. (2017). Multifunctions of Dual Zn/Mg Ion Co-implanted Titanium on Osteogenesis, Angiogenesis and Bacteria Inhibition for Dental Implants. Acta Biomater. 49, 590–603. 10.1016/j.actbio.2016.11.067 27915020

[B46] Zhang X. He Y. Huang P. Jiang G. Zhang M. Yu F. (2020). A Novel Mineralized High Strength Hydrogel for Enhancing Cell Adhesion and Promoting Skull Bone Regeneration *In Situ* . Composites Part B: Eng. 197, 108183. 10.1016/j.compositesb.2020.108183

[B47] Zhang X. Huang P. Jiang G. Zhang M. Yu F. Dong X. (2021). A Novel Magnesium Ion-Incorporating Dual-Crosslinked Hydrogel to Improve Bone Scaffold-Mediated Osteogenesis and Angiogenesis. Mater. Sci. Eng. C. 121, 111868. 10.1016/j.msec.2021.111868 33579495

[B48] Zhang Y. Chen M. Tian J. Gu P. Cao H. Fan X. (2019). *In Situ* bone Regeneration Enabled by a Biodegradable Hybrid Double-Network Hydrogel. Biomater. Sci. 7, 3266–3276. 10.1039/c9bm00561g 31180391

[B49] Zhang Y. Xie R. L. Croce C. M. Stein J. L. Lian J. B. van Wijnen A. J. (2011). A Program of microRNAs Controls Osteogenic Lineage Progression by Targeting Transcription Factor Runx2. Proc. Natl. Acad. Sci. U S A. 108, 9863–9868. 10.1073/pnas.1018493108 21628588 PMC3116419

