# Correction: Biomineralized composite liquid crystal fiber scaffold promotes bone regeneration by enhancement of osteogenesis and angiogenesis

**DOI:** 10.3389/fphar.2025.1617849

**Published:** 2025-06-16

**Authors:** Yi Zhan, Bing Deng, Huixian Wu, Changpeng Xu, Ruiying Wang, Wenqiang Li, Zhixiong Pan

**Affiliations:** ^1^ Department of Orthopedic Surgery, Affiliated Hospital of Guilin Medical University, Guilin, China; ^2^ Department of Orthopaedics, Guangdong Second Provincial General Hospital, Guangzhou, China; ^3^ Engineering Technology Research Center for Sports Assistive Devices of Guangdong, Guangzhou Sport University, Guangzhou, China; ^4^ Guangxi Health Commission Key Laboratory of Basic Research in Sphingolipid Metabolism Related Diseases, The Affiliated Hospital of Guilin Medical University, Guilin, China

**Keywords:** liquid crystal fiber, biomimetic mineralization, osteogenic differentiation, vascularization, regeneration

In the published article, there was an error in Figure 6A as published. It is because images from different groups were stored in the same folder during image shooting, resulting in the misuse of images. The corrected Figure 6A and its caption appear below.

**FIGURE 6:**
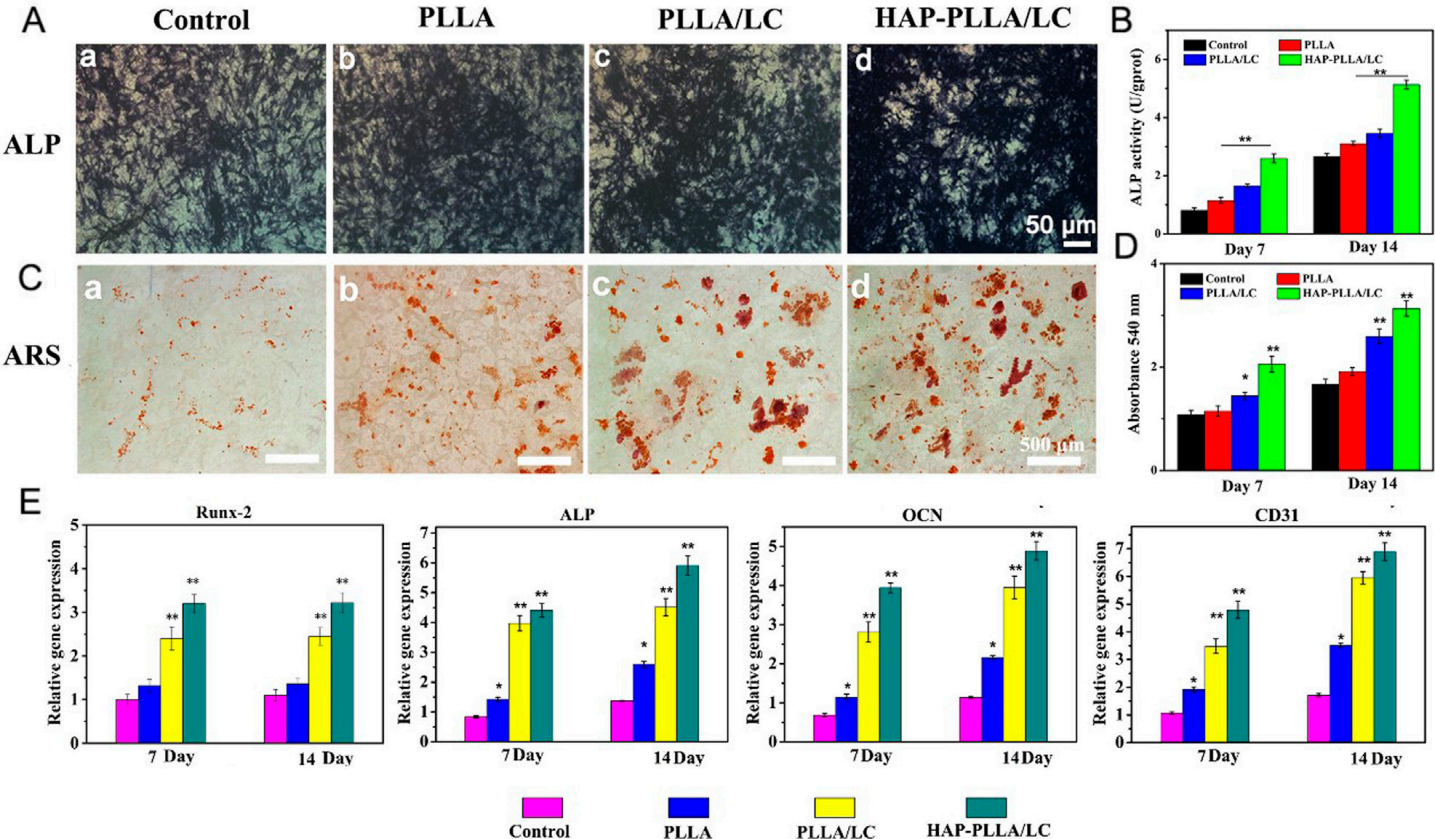
**(A)** Alkalinephosphatase (ALP) staining and **(B)** its quantitative analysis of BMSC cells cultured on the scaffolds for 14 days. **(C)** Alizarin red (AR) staining and **(D)** its quantitative analysis of BMSC cells cultured on a culture plate, PLLA, PLLA/LC, and HAP-PLLA/LC fiber scaffold for 14 days, respectively. **(E)** Real-time quantitative PCR (RT-qPCR) analysis of osteogenic and vascularized-related gene expression of cells after culturing for 7 and 14 days.

The authors apologize for this error and state that this does not change the scientific conclusions of the article in any way. The original article has been updated.

